# Microbiota-Derived Short-Chain Fatty Acids Boost Antitumoral Natural Killer Cell Activity

**DOI:** 10.3390/jcm13133885

**Published:** 2024-07-02

**Authors:** Marina Pérez, Berta Buey, Pilar Corral, David Giraldos, Eva Latorre

**Affiliations:** 1 Departamento de Bioquímica y Biología Molecular y Celular, Facultad de Ciencias, Universidad de Zaragoza, 50009 Zaragoza, Spain; 2 Departamento de Farmacología, Fisiología y Medicina Legal y Forense, Facultad de Veterinaria, Universidad de Zaragoza, 50013 Zaragoza, Spain; 3 Instituto de Investigación Sanitaria de Aragón (IIS Aragón), 50009 Zaragoza, Spain; 4 Instituto Agroalimentario de Aragón (IA2), 50013 Zaragoza, Spain

**Keywords:** microbial metabolites, SCFAs, multiple myeloma, immunotherapy, cancer, postbiotics

## Abstract

**Background:** The intestinal microbiota can regulate numerous host functions, including the immune response. Through fermentation, the microbiota produces and releases microbial metabolites such as short-chain fatty acids (SCFAs), which can affect host homeostasis. There is growing evidence that the gut microbiome can have a major impact on cancer. Specific gut microbial composition and metabolites are associated with tumor status in the host. However, their effects on the antitumor response have scarcely been investigated. Natural killer (NK) cells play an important role in antitumor immunity due to their ability to directly identify and eliminate tumor cells. **Methods:** The aim of this study was to investigate the effects of SCFAs on antitumoral NK cell activity, using NK-92 cell line. **Results:** Here, we describe how SCFAs can boost antitumoral NK cell activity. The SCFAs induced the release of NK extracellular vesicles and reduced the secretion of the anti-inflammatory cytokine IL-10. The SCFAs also increased the cytotoxicity of the NK cells against multiple myeloma cells. **Conclusions:** Our results indicate, for the first time, the enormous potential of SCFAs in regulating antitumoral NK cell defense, where modulation of the SCFAs’ production could play a fundamental role in cancer immunotherapy.

## 1. Introduction

The intestinal microbiota, a diverse community of microorganisms that colonize our gastrointestinal tract, has been shown to be a crucial element in regulating the health of the host. Their influence extends beyond the digestion and absorption of nutrients to the regulation of fundamental processes in the body, such as the maintenance of homeostasis and immune responses [1]. In fact, symbiosis between the host and microbiota is crucial for the proper functioning of the immune system, so its imbalance can contribute to various disorders and chronic diseases such as cancer, inflammatory bowel disease, or diabetes [2].

Mutual interactions between the microbiota and immune system appear to be a key factor contributing significantly to cancer immune responses [3]. In general, a more diverse gut microbiome has a positive effect on the functional diversity of the immune system, which is likely to reduce the risk of developing cancer [4]. Recently, microbial community diversity has been described as an independent predictor of survival in cervical cancer [5].

A key aspect of host–microbiome crosstalk is determined by a variety of microbial metabolites, which may have direct effects not only in the gut but also in distant organs [6]. Several studies have detected a plethora of microbial molecules in the human bloodstream, and it is estimated that between 5 and 10% of all plasma metabolites are derived from the gut microbiota [7].

Short-chain fatty acids (SCFAs) are among the most representative and abundant microbial metabolites. These compounds are produced by the microbiota through the fermentation of indigestible dietary fiber and appear to have a significant impact on the modulation of the immune response [8]. SCFAs alleviate allergic airway inflammation by inducing the differentiation of T cells into regulatory T cells (Treg) [9]. Similarly, SCFAs can regulate the size and function of the colonic Treg pool and protect against colitis [10]. SCFAs have also shown critical effects on immune cell migration and apoptosis [11], inflammasome responses [12], and antibody production [13]. In addition, SCFAs can promote dendrite elongation by inhibiting histone deacetylases (HDACs), resulting in increased antigen uptake and presentation in dendritic cells [14]. By regulating the HDACs, SCFAs can influence not only dendritic cells but also monocytes [15], neutrophils [16], and macrophages [17].

SCFAs such as acetate, propionate, and butyrate are microbial metabolites with diverse biological functions. These three SCFAs are found in the highest concentrations in the large intestine (approximately 50 mM), but they are also found in peripheral tissues, with acetate present at approximately 50 μM [18]. While the highest concentrations are found in the colon, the presence of SCFAs in peripheral tissues suggests that they may also play a regulatory role outside the gastrointestinal tract. Plasma acetate concentrations can be much higher (1–6 mM), especially in individuals who consume a high-fiber diet. Acetate concentrations in the cecum and serum correlate negatively with airway hyper-responsiveness and with the number of eosinophils in the bronchoalveolar fluid of mice with asthma [9]. SCFAs are known to have anti-inflammatory properties. Studies have shown that butyrate can inhibit NF-κB activation in intestinal epithelial cells, immune cells, and other cell types in the gut [19]. By blocking NF-κB signaling, butyrate can reduce the expression of pro-inflammatory cytokines and chemokines [20] and promote the expression of anti-inflammatory factors [21]. Similarly, acetate and propionate have well-documented anti-inflammatory effects through suppressing inflammatory cytokines [22].

Gut dysbiosis occurs when the balance between the microbiota and the host is disturbed and manifests as changes in taxonomic composition, metabolic products, and secretory vesicles, all of which have been linked to a wide range of diseases, including cancer [23]. Available evidence from animal models has shown that microbes can facilitate the initiation and progression of various types of cancer including gastric cancer, colorectal cancer, hepatocellular carcinoma, breast cancer, or pancreatic ductal adenocarcinoma [24].

Recent studies have suggested that the antitumor activity of immune cells may be modulated by the gut microbiota and its metabolites, potentially offering benefits for cancer management [4]. Gut dysbiosis leading to altered SCFA production has been linked to cancer progression [9]. Several studies have found significant differences in the composition of the gut microbiota between patients with colorectal cancer and healthy controls [25], where butyric-producing bacteria and lactic-acid-producing bacteria are under-represented [26]. Similarly, 21 bacterial strains are significantly enriched in patients with gastric carcinoma and *Porphyromonas*, *Streptococcus*, *Bifidobacteria*, and *Fusobacteria* are over-represented in pancreatic cancer patients [27]. However, the underlying mechanism by which the microbiota influence cancer progression is unclear. 

Immunotherapy, which focuses on enhancing patients’ immune response to eliminate tumor cells, has gained prominence in cancer research. Natural killer (NK) cells are particularly noteworthy for their ability to directly identify and eliminate tumor cells [28]. NK cell-based immunotherapies are attracting increasing interest in the field of cancer treatment. Actually, NK cells have proven an effective cancer immunotherapy tool, and NK cell therapy has entered phase I/II clinical trials [29]. Recent developments have greatly increased the therapeutic potential of NK cells by providing them with enhanced recognition and cytotoxic capacities. Therefore, the aim of the present study is to investigate the potential effects of SCFAs, acetate, propionate, and butyrate on antitumor NK cell activity.

## 2. Material and Methods

### 2.1. Cell Lines

NK-92 cells are a highly cytotoxic, IL-2-dependent, and CD16-negative cell line that was first isolated from a 50-year-old patient with rapidly progressive non-Hodgkin’s lymphoma and is currently used as an immortalized cell model for NKs [30]. The NK-92 cell line RRID:CVCL_2142 was generously donated by Dr Julián Pardo (Centro de Investigación Biomédica de Aragón, Zaragoza, Spain). The NK-92 cells were cultured in an α-MEM (Minimal Essential Medium) containing 12.5% FBS (fetal bovine serum), 12.5% HS (horse serum), 1% glutamax, and 1% antibiotics (penicillin 100 U/mL and streptomycin 100 μg/mL). In addition, 25 IU/mL of the IL-2 was added to the cell culture medium. For cultivation, this cell line was diluted 1/3 every 48 h to maintain the cells between 2 and 6 × 10^5^ cells/mL.

MM.1S cells (RRID:CVCL_8792) are a cell model of multiple myeloma and were purchased from ATCC (CRL-2974). This cell line originated from the MM.1 cell line obtained in 1986 from the peripheral blood of a 42-year-old woman [31]. RPMI 1640 GlutaMAX^TM^ medium supplemented with 10% FBS and 1% antibiotics (penicillin 100 U/mL and streptomycin 100 μg/mL) was used for the cell culture. Subcultures were performed every 72 h at a density of 2 × 10^5^ cells/mL. All reagents were provided by Life Technologies (Paisley, UK). 

### 2.2. Cell Proliferation

An MTT assay was used to measure cellular metabolic activity as an indicator of cell proliferation [32]. A total of 1.5 × 10^5^ NK-92 cells/mL were seeded and treated with 10 μM acetate, 2 μM butyrate, or 2 μM propionate for 48 h. After treatment, the cells were incubated with 15 μL of the MTT for 2 h and then centrifuged. In addition, 100 μL of acidified isopropanol was added to dissolve the formazan crystals. The absorbance was measured at 550 nm with a spectrophotometer. The results are expressed as percentages of the control.

### 2.3. Extracellular Vesicles Study

To visualize the extracellular vesicles (EVs) after 48 h of treatment with SCFAs, the NK-92 cells were stained with two fluorescent probes. A Hoechst 33342 probe was used to label the nuclei and CFSE (carboxyfluorescein succinimidyl ester) was used for the cytoplasm. For this purpose, 1.5 × 10^5^ NK-92 cells/mL were seeded with 1 μM of the CFSE along with the SCFAs and incubated for 48 h. The cells were labeled with 2 μg/mL of the Hoechst 33342 and observed under a fluorescence microscope. Three photographs were taken from different fields for each sample. 

To quantify the EVs, the protein content of the supernatant was measured using a BCA assay. A total of 1.5 × 10^5^ NK-92 cells/mL were seeded with the indicated concentrations of each SCFA and incubated for 48 h. Then, the supernatant was centrifuged (2766× *g* for 20 min), and 0.22-micron filtration was performed to ensure that all the cells were removed. Finally, the supernatants were sonicated, and protein levels were measured using a BCA assay (Thermo Scientific™) (Rockford, IL, USA).

### 2.4. Cytokines Array

A human cytokine antibody array (ab133996) from Abcam (Amsterdam, The Netherlands) was used according to the manufacturer’s instructions. A total of 1 × 10^6^ NK cells per well were seeded in a 6-well plate and treated with the SCFAs as previously described. Then, the supernatants were centrifuged at 2766× *g* for 20 min, and 0.22-micron filtration was performed. Briefly, after blocking the array membranes, 1 mL of the supernatant from each condition was added to the membranes and incubated overnight at 4 °C on a rocking shaker. Following 4 washes in wash buffer I and 3 washes in wash buffer II, the membranes were incubated for 2 h at room temperature, first with biotin-conjugated anti-cytokines and then with HRP-conjugated streptavidin. Finally, the washed arrays were treated with chemiluminescence detection reagents, and images were acquired using an Amershan Imager 600 (GE Healthcare, Piscataway, NJ, USA).

### 2.5. Cytotoxicity Assay 

The NK-92 cells were seeded at 1.5 × 10^5^ cells/mL, treated with the SCFAs (10 μM acetate, 2 μM butyrate, 2 μM propionate) for 48 h, and cocultured with the multiple myeloma cells (MM.1S) at a 1:1 effector/target ratio. The cytotoxicity assay for the NK-92 cells consisted of confronting 5 × 10^4^ myeloma cells and 5 × 10^4^ NK-92 cells. In addition, we exposed 5 × 10^4^ myeloma cells to 100 μL of the supernatant from the SCFA-treated NK cells after 24 and 48 h of treatment. In both cases, the cells were cocultured for 4 h to ensure their cytotoxic effect (based on previous experiments in our research group). After this time, labeling was carried out with DY634 Annexin V. In addition, the NK-92 cells were labeled with a green fluorescent protein so that the NK cells could differentiate from the myeloma cells. All stained cells were measured using a FACSCalibur flow cytometer (BD Biosciences, San Jose, CA, USA), and the data were analyzed using FlowJoTM v7.0 Software (BD Life Sciences, Ashland, OR, USA).

### 2.6. Statistical Analysis

All results are expressed as means ± the standard deviation (SD). Statistical comparisons between the untreated (control) and treated were performed using two-tailed unpaired *t*-tests. Statistical significance was set at *p* < 0.05. Statistical analysis was carried out with the computer-assisted Prism GraphPad Program v9 (GraphPad Software, Boston, MA, USA).

## 3. Results

### 3.1. SCFA Butyrate Increases NK Cell Proliferation

**Figure 1:**
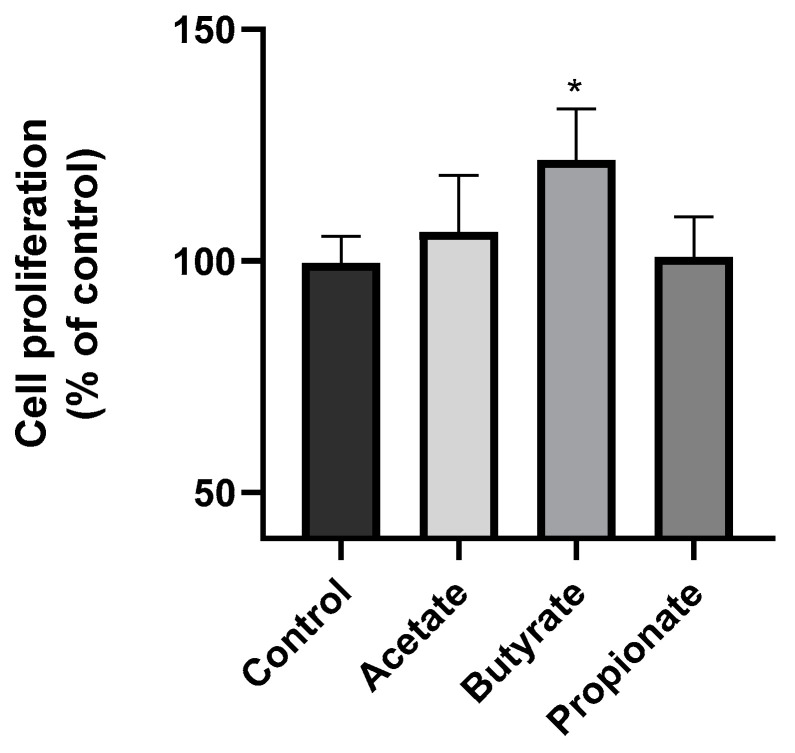
Natural killer (NK) cell proliferation. A total of 1.5 × 10^5^ NK-92 cells/mL were seeded and treated with 10 μM acetate, 2 μM butyrate, or 2 μM propionate for 48 h. The results are expressed as the mean ± SD of the 5 independent experiments (n = 10). * *p* < 0.05.

First, we investigated the potential impact of SCFAs on the proliferation of NK cells. To achieve this objective, we conducted an MTT assay in which the NK cells were subjected to the SCFA treatment for 48 h. The results of this experiment are depicted in Figure 1. Notably, our findings revealed a significant effect of the butyrate on NK cell proliferation, with a remarkable increase of 20% observed relative to that in the control group. However, our analysis indicated that neither the acetate nor propionate had any discernible impact on the growth of the NK cells after 48 h of treatment. 

### 3.2. SCFAs Affect NK Cell Secretome

**Figure 2:**
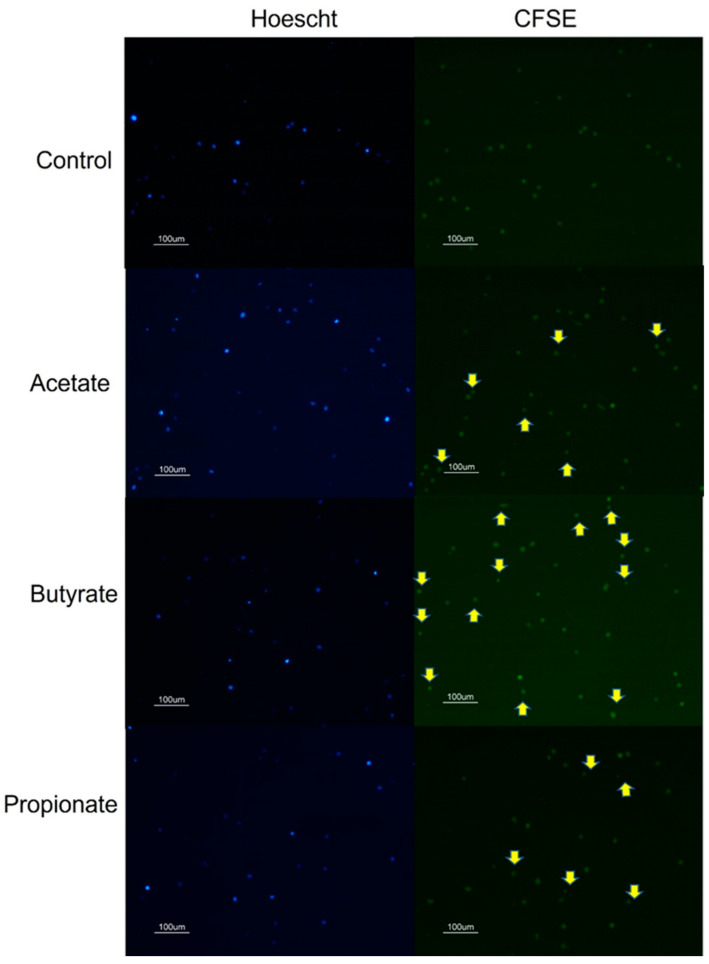
NK extracellular vesicle release. NK-92 cells after 48 h of treatment with short-chain fatty acids (SCFAs) (10 μM acetate, 2 μM butyrate, or 2 μM propionate) were stained with Hoescht 33342 for nuclei visualization and CFSE for the cytoplasm and observed under a fluorescence microscope. Three photographs of different fields were taken for each sample of three independent experiments (n = 9). Yellow arrows indicate Hoescht 33342-negative/CFSE-positive vesicles.

**Figure 3:**
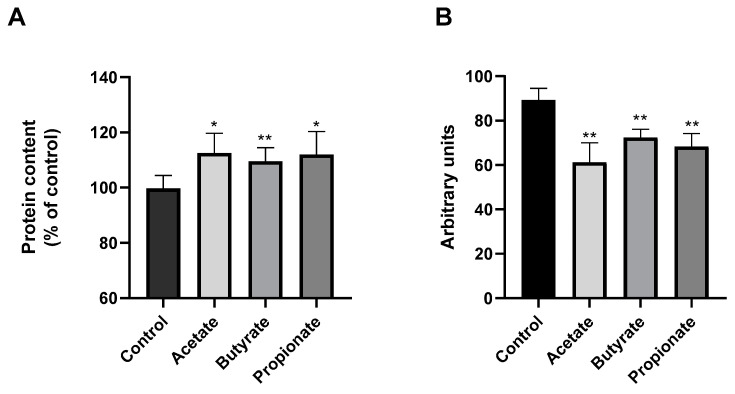
(**A**) Protein content. Protein content of the supernatants was measured by a BCA assay. A total of 1.5 × 10^5^ NK-92 cells/mL were seeded and incubated for 48 h with 10 μM acetate, 2 μM butyrate, or 2 μM propionate. The results are expressed as the mean ± SD of 3 independent experiments (n = 9). * *p* < 0.05 ** *p* < 0.01. (**B**) IL-10 release by NK cells. A total of 5 × 10^5^ NK-92 cells/mL were seeded and treated with 10 μM acetate, 2 μM butyrate, or 2 μM propionate for 48 h; then, the supernatants were analyzed by a cytokine array. The results are expressed as the mean ± SD of 3 independent experiments (n = 6). ** *p* < 0.01.

In this study, we explored the influence of SCFAs on the secretome of NK cells. All experiments were conducted following a 48h treatment with the SCFAs. We analyzed the release of NK-EVs by the Hoechst 33342/CFSE labeling. The size of the detected Hoechst 33342-negative/CFSE-positive vesicles was comparable to that of the previously described NK-EV [33]. Our results showed that the SCFAs, acetate propionate, and butyrate induced extracellular vesicle release by the NK cells, as shown in Figure 2. To quantify the NK-EV release, the protein content of the supernatant was measured. Figure 3A shows that all three of the SCFAs increased the protein content of the NK supernatants in a similar manner. 

Then, we analyzed the cytokines released by the NK cells after 48 h treatment with the three SCFAs. Our analysis of the NK cell secretome involved examining the release of various cytokines, including G-CSF, GM-CSF, GRO, GRO-α, IL-1α, IL-2, IL-3, IL-5, IL-6, IL-7, IL-8, IL-10, IL-13, IL-15, IFN-γ, MCP-1, MCP-2, MCP-3, MIG, RANTES, TGF-β1, TNF-α, and TNF-β, in response to the SCFA treatment. Intriguingly, significant changes were observed only in the release of interleukin-10 (IL-10). As shown in Figure 3B, the SCFAs elicited a notable reduction in IL-10 release by the NK cells.

### 3.3. SCFAs Enhance the Antitumor Cytotoxicity of NK Cells

**Figure 4:**
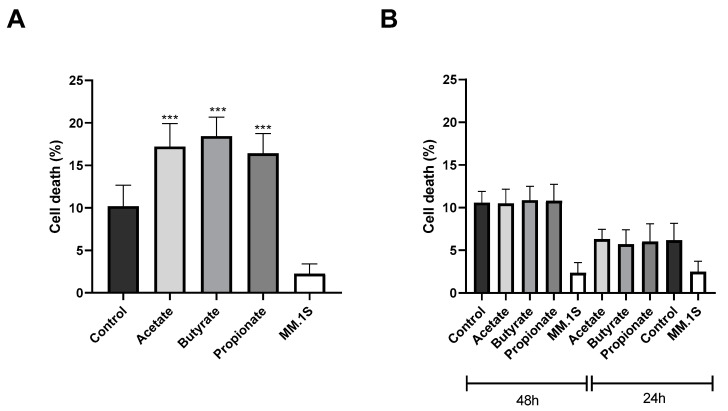
(**A**) SCFA effects on NK cells’ antitumor cytotoxicity. A total of 5 × 10^4^ NK cells, pre-treated with SCFAs for 48 h, were then challenged with multiple myeloma cells (MM.1S) at a 1:1 (effector/target) ratio for 4 h. Control: MM.1S + untreated NK cells. Acetate, butyrate, and propionate: MM.1S + SCFA-treated NK cells. MM.1S: unexposed to NK cells. (**B**) Effect of SCFA-treated NK supernatant on multiple myeloma cell death. A total of 100 μL of supernatant from NK cells treated with SCFAs for both 24 and 48 h were added to 5 × 10^4^ myeloma cells and incubated for 4 h. Labeling was carried out with DY634 Annexin V and analyzed by flow cytometry. The results are expressed as the mean ± SD of 3 independent experiments (n = 9). *** *p* < 0.001.

We also tested the effects of the SCFAs on the antitumor activity of the NK cells by using a myeloma cell line (MM.1S) as a cancer cell model. For this purpose, the NK-92 cells were pretreated with the SCFAs for 48 h prior to coculturing with the target cells at a 1:1 (effector/target) ratio and incubated at 37 °C for 4 h. Subsequently, cell death in the myeloma cell population was analyzed by flow cytometry. The results showed that all three of the SCFAs enhanced the antitumor cytotoxicity of the NK cells. Specifically, the acetate increased myeloma cell death by 7%, the butyrate by 8%, and the propionate by 6% compared to those in the control group (Figure 4A). 

To discern whether the heightened cytotoxicity of the NK cells resulted from a direct or indirect effect of the NK cells, we evaluated the impact of the supernatant from the SCFA-treated NK cells on myeloma cell death by exposing the myeloma cells to the supernatant for 4 h. Notably, the supernatant was collected from the NK cells treated with the SCFAs for both 48 and 24 h. This decision was made due to the lack of observed effects after 48 h of treatment, aiming to explore whether a shorter treatment duration could elicit any discernible effects. However, as Figure 4B shows, the supernatant failed to induce any significant change in myeloma cell death after 24 or 48 h SCFA treatment. This suggests that the observed increase in the NK cell cytotoxicity is likely attributed to a direct effect of the NK cells rather than an indirect effect mediated by soluble factors released by the SCFA-treated NK cells.

## 4. Discussion

There is growing evidence that the gut microbiome and its metabolites can have a major impact on tumorigenesis, prognosis, and treatment outcomes [34]. Specific gut microbial composition and metabolites are associated with tumor status in the host. Interventions targeting the gut microbiota may confer a protective effect against tumors by manipulating its structure and associated metabolites. In this context, microbiota-derived SCFAs have been shown to exert significant immunoregulatory effects. However, there are still gaps in our knowledge regarding the specific mechanisms through which SCFAs can regulate the immune system. Here, the aim was to investigate the effects of SCFAs on NK cells. This study opens the door to exploring the potential benefits of SCFAs on the antitumoral activity of NK cells. 

Our results show, for the first time, that the SCFAs butyrate, propionate, and acetate affect NK cells. Specifically, butyrate appears to increase NK cell proliferation. In agreement with our results, butyrate has also been proven to increase proliferation by inhibiting p38 MAPK and JNK signaling pathways in other cells, such as intestinal cells [35] and human neural progenitor cells [36]. In fact, butyrate has been shown to regulate cell proliferation in in vitro and in vivo models [37].

NK cells are critical elements in the antitumor immune response, and their effector functions are tightly controlled by a complex network of activating and inhibitory receptors. There is little information in the literature on the cytokines, chemokines, and growth factors secreted by NK cells. However, the NK secretome could have a critical impact, not only on cancer cell death but also on tumor growth [38]. Our results have shown that SCFAs can alter the NK secretome, and thus directly influence the functions of NK cells. On the one hand, SCFAs seem to induce the release of NK-EVs. The NK-EVs could carry cargo such as cytotoxic proteins, microRNAs, or cytokines and use multiple mechanisms to kill tumor cells or exert immunomodulatory activity [33]. In this sense, there is increasing evidence that NK-EVs could be involved in the antitumor activity of NK cells [39]. Previous studies have linked NK-derived vesicles to melanoma cytotoxicity via Fas ligand and perforin expression [40,41]. However, our study revealed that the SCFA-treated NK cells increased extracellular vesicle release, although they did not exert cytotoxic effects on multiple myeloma cells. Notably, the contents of these vesicles remain uncharacterized, highlighting the necessity for further exploration into their molecular composition. In contrast, we found that the SCFAs not only increased NK-EV secretion but also exerted dual effects by reducing the secretion of the anti-inflammatory cytokine IL-10. Although our analysis encompassed the secretion of 23 cytokines, significant alterations were observed only in terms of IL-10 release. IL-10 is a multifunctional cytokine with both immunosuppressive and antiangiogenic functions. Among other effects, IL-10 promotes tumor cell proliferation and metastasis via immunosuppression [42]. The neutralization of IL-10 induces a tumor-specific cytotoxic immune response [27]. In agreement with our results, the butyrate supplementation promotes the expression of antitumor cytokines in cytotoxic T cells [43].

Elucidating the dynamic nature of NK cells holds great potential for cancer immunotherapy. Many factors influence the phenotype and function of NK cells [44]. Intrinsic factors, such as metabolic flux [45], and extrinsic factors, such as the tumor microenvironment [46], cooperatively define NK cytotoxicity. It is in the tumor microenvironment that the role of the microbiota and their metabolites as potential regulators of NKs stands out. Interestingly, cytokines present in the tumor microenvironment may act as modulators of NK function [47]. The upregulation of IL-10 expression induces the dysregulation of the NK cell surface receptors, especially the inhibitory receptor NKG2A, which displays a depletion phenotype [48]. In addition, IL-10 decreases MHC expression in tumors, contributing to less destruction by the NK cells [49]. In agreement, our results show that SCFAs induce a reduction in IL-10 release by the NK cells to counteract its pro-tumorigenic effects.

Increasing evidence suggests that SCFAs can influence carcinogenesis [50]. SCFAs enhance IFNγ-mediated responses and improve the capacity to differentiate T cells into cytotoxic memory cells [51]. Although we observed that the NK supernatant did not induce cytotoxic effects, our results showed that the SCFAs could increase NK cell cytotoxicity in multiple myeloma cells. This disparity may stem from the complex interplay between the SCFAs and the NK cells. Direct SCFA treatment could induce intracellular changes in the NK cells, thereby enhancing their cytotoxic potential. Nevertheless, the absence of cytotoxic effects in the supernatant implies that factors influencing direct cellular interactions may not be fully mirrored in the isolated vesicle content. 

NK cells play a central role against multiple myeloma. In fact, NK cells can induce cell clusters that facilitate NK and T cells’ anti-myeloma activity [52]. Recently, SCFAs and the gut microbiota associated with their production have been shown to have beneficial effects on multiple myeloma evolution and response to treatment. Bacteria involved in SCFA production, including *Prevotella*, *Blautia*, *Weissella*, and *Agathobacter*, were more prevalent in premalignant or complete remission cancer samples. Patients with higher levels of *Agathobacter* had better overall survival. The serum levels of butyrate and propionate decrease with myeloma progression, and butyrate is positively associated with a better response [53]. 

In support of our results, some studies have shown that SCFAs reinforce the immune antitumoral defense. Acetate increases the antitumor response of the T cells in breast cancer progression [54], and propionate suppresses leukemia progression both in vivo and in vitro models [55]. Tumor cells treated with SCFAs induced much greater activation of the T cells than untreated cells [56]. SCFAs have also shown potential for sensitizing tumor cells to immune checkpoint inhibitors in vitro [57] and in patients [58]. 

In addition to SCFAs, various microbiota-derived metabolites such as secondary bile acids, oligosaccharides, tryptophan metabolites, inosine, and polyamines also appear to be able to influence tumor immunity [59]. Indeed, the tryptophan metabolites produced by *Lactobacillus* can activate the aryl hydrocarbon receptor in tumor-associated macrophages, which inhibits intratumoral T-cell infiltration in pancreatic ductal adenocarcinoma [60]. In contrast, deoxycholic acid, a secondary bile acid, appears to contribute to the malignant transformation of the intestinal epithelium [61].

The potential of microbial strategies for cancer therapy is being demonstrated in many clinical trials. Specifically, the human microbiota could be modified to enhance the host response to existing anticancer therapies, minimize associated adverse toxicity, and reduce drug resistance in immunotherapy, chemotherapy, cancer surgery, and radiotherapy. Specific interventions targeting the microbiota include, but are not limited to, nutritional interventions, prebiotics, probiotics, postbiotics, targeted antibiotic approaches, and fecal microbiota transplantation. Fecal microbiota transplantation seems to enhance immunotherapy efficacy and mitigate immune-related adverse events in cancer [62], being a promising approach under clinical investigation. Actually, a search of the term fecal microbiota transplantation showed seven active clinical trials on renal cell carcinoma (ClinicalTrials.gov identifiers: NCT04163289; NCT04758507), melanoma (ClinicalTrials.gov identifier: NCT03341143), gastrointestinal cancers (ClinicalTrials.gov identifiers: NCT04729322; NCT04130763), and other solid tumors (ClinicalTrials.gov identifiers: NCT03686202; NCT03838601).

The microbiota and its metabolic products, such as SCFAs, represent a fascinating area of research that continues to reveal their key influence on immune responses and their determining role in cancer. The intake of SCFA-producing microbes or SCFA treatment before or during treatment with anticancer drugs in colorectal cancer may induce the therapeutic efficacy of anticancer drugs by controlling epigenetic modification regulation, immune cell activation, and gene expression regulation [63]. Similarly, SCFAs are expected to become important alternatives for their synergistic anticancer effects and reduction in drug resistance in breast cancer [64] and cervical cancer [65].

### Limitations of the Study and Future Perspectives

Our investigation into the effects of SCFAs on NK cells underscores their potential role in cancer immunotherapy. Specifically, acetate, butyrate, and propionate increased NK-92 cell cytotoxicity against the myeloma cell line and the release of extracellular vesicles, while also reducing the secretion of IL-10. This suggests a potential enhancement of NK cell antitumor activity. Notably, only butyrate demonstrated an additional effect by increasing the NK-92 cell proliferation, highlighting the multifaceted impact of SCFAs on NK cell function. However, our study is not without limitations. We primarily focused on in vitro research, which may not fully capture the complexities of the tumor microenvironment. The SCFA concentrations used in this study were similar to those described for plasma; it would be interesting to explore the potential effects of higher concentrations.

While our findings provide valuable insights, further research is needed to elucidate the underlying molecular mechanisms and validate these findings in animal cancer models. Additionally, this study has been carried out with the NK-92 cell line and our results may differ from using primary NKs. Therefore, further studies should explore the potential of SCFAs on the primary NKs.

## 5. Conclusions

Our study demonstrates that acetate, propionate, and butyrate increase the NK cytotoxicity when challenged with myeloma cells. The SCFAs also increase the release of NK-EVs, but there are no displayed cytotoxic effects. In addition, the SCFAs reduce the IL-10 release by the NKs, potentially affecting the tumor microenvironment. 

NK cell-based therapies are emerging as effective treatments for cancers. Their research has grown exponentially and currently constitutes a major area of immunotherapy innovation. The next generation of NK cell products is focused on enhancing activating signals and proliferation, suppressing inhibitory signals, and promoting their homing to tumors. In this context, SCFAs could be a novel approach to enhance NK cell cytotoxicity.

## Data Availability

The data that support the findings of this study are available from the corresponding author (E.L), upon reasonable request.
